# 3D‐Analysis of Peri‐Implant Soft Tissue Gain With Collagen Matrix and Connective Tissue Graft: A Randomized Control Trial

**DOI:** 10.1111/cid.70043

**Published:** 2025-04-20

**Authors:** Igor Ashurko, Svetlana Tarasenko, Mary Magdalyanova, Maxim Balyasin, Anna Galyas, Sabina Kazumyan, Nadiya Safi, Alexey Unkovskiy

**Affiliations:** ^1^ Department of Dental Surgery Sechenov First Moscow State Medical University Moscow Russia; ^2^ Department of Prosthodontics, Geriatric Dentistry and Craniomandibular Disorders Charité—Universitätsmedizin Berlin Berlin Germany

**Keywords:** clinical research, implantology, randomized controlled trial, soft tissue grafting

## Abstract

**Objectives:**

To compare the efficacy of the connective tissue graft (SCTG) and the collagen matrix (VXCM) in terms of soft tissue gain at the buccal site around a single implant.

**Methods:**

The study was designed as a randomized, controlled clinical trial. This trial was registered in ClinicalTrial.gov with the identifier NCT05870774 and is accessible under the following link: https://clinicaltrials.gov/study/NCT05870774?term=NCT05870774&rank=1. This clinical trial was not registered prior to participant recruitment and randomization. Patients with a single tooth gap and horizontal soft tissue thickness deficiency were enrolled in the study. Sites were randomly allocated to the control (SCTG) or test group (VXCM: Geistlich Fibro‐Gide, Geistlich Pharma AG, Wolhusen, Switzerland) to augment buccal soft tissue thickness. The primary outcome was soft tissue gain 3 months post‐op. Secondary outcomes included soft tissue gain 6 months post‐op, the pink aesthetic score (PES), and patient‐reported outcome measures (PROMs).

**Results:**

Patients' recruitment started on 28 October 2021 and ended on 25 December 2022. Thirty‐two patients were enrolled and subjected to intervention. Sixteen patients were included per group. Three months post‐op, soft tissue gain at the buccal site was 1.77 ± 0.61 mm in the VXCM group and 1.26 ± 0.41 mm in the SCTG group (*p* = 0.0003). Six months post‐op, soft tissue gain was 1.11 ± 0.44 mm in the VXCM group and 1.43 ± 0.81 mm in the SCTG group (*p* = 0.0459). PROMs, including pain perception, favored the VXCM group. SCTG demonstrated favored results in PES.

**Conclusion:**

SCTG remains the gold standard for increasing soft tissue thickness in terms of the clinical result.

## 1 Introduction

Along with the evolution of surgical and prosthetic treatment strategies using dental implants, patient expectations are also increasing. Current aesthetic requirements of patients are determined not only by the shape and color of implant restorations, but also by the alveolar ridge architecture, texture, and color of the surrounding soft tissues [1, 2, 3].

It has long been recognized that the loss of the buccal contour is an inevitable consequence following the extraction of a tooth [4, 5]. The extent of these changes is clinically significant because they create a distinct buccal concavity of the alveolar ridge that can complicate aesthetic rehabilitation [6]. Besides, there is an understanding of the impact of soft tissue thickness in the implant area on the marginal bone stability, peri‐implant soft tissue color, and the overall aesthetic component of the treatment [3, 7, 8]. Threshold values for both vertical and horizontal thickness of soft tissues have been established [9, 10, 11, 12, 13]. There is undeniable evidence that soft tissue is the key to maintaining peri‐implant health. Thus, long‐term clinical studies show stable results of treatment with dental implants after 7–12 years, even in the absence of buccal bone in the implant area, but with adequate soft tissue conditions [14, 15].

Historically, autogenous soft tissue grafts were the first graft materials to be used in reconstructive surgeries around teeth and implants [16, 17]. It was determined that augmentation using the autogenous connective tissue graft or their collagen analogues allows compensation for buccal invagination, as well as to increase soft tissue thickness, which is important from the point of view of achieving favorable conditions for implant health and ensuring aesthetic results [18, 19].

The use of a subepithelial connective tissue graft (SCTG) harvested from the hard palate or maxillary tuberosity is still considered the “gold standard” for soft tissue augmentation around dental implants [20, 21, 22]. However, postoperative discomfort at the donor site has motivated researchers to develop alternative graft substitute materials [23, 24]. Currently, in clinical practice, xenogenic collagen matrices are more commonly employed, having demonstrated efficacy in preclinical and clinical studies [25, 26]. The use of matrices significantly expedites the time required for surgical intervention and reduces postoperative discomfort compared to autogenous grafts, which are among the primary advantages of these materials. Among the most prevalent are matrices based on uncrosslinked collagen, which possess a natural structure (referred to as bilayer matrices). Numerous studies have been published, showcasing favorable clinical outcomes from the use of such matrices for augmenting the thickness of soft tissues and correcting deformities in the buccal contour of the alveolar ridge [21, 22, 27, 28, 29, 30].

An increasing number of publications has recently been published describing the use of VXCM, which has demonstrated promising results in preclinical and clinical studies [26, 31, 32, 33, 34]. However, it remains unclear whether soft tissue augmentation with VXCM is inferior to the application of SCTG in terms of mucosal thickening in the area of single implants, as the available randomized controlled trials present conflicting data. Another important limitation is that systematic reviews and meta‐analyses typically include the analysis of matrices without considering their structural characteristics [20, 35, 36].

It should also be noted that the timing of soft tissue grafting also plays an important role *in* the overall efficiency of soft tissue augmentation. Thus, according to the clinical review described by L. Manchini (2023), before/after implant placement or at healing abutment installation are considered to be the gold standard time points of soft tissue augmentation around implants [37]. Nevertheless, modern trends, including patient expectations, are aimed at reducing the treatment time and the number of surgeries. On the one hand, soft tissue augmentation in single‐stage implantation seems to be less predictable due to different healing approaches; however, there is a lack of data regarding the use of cross‐linked collagen matrices in such an approach, which is undoubtedly of interest.

The primary objective of this study was to compare VXCM with SCTG, applied in combination with a single‐stage implantation in a randomized control trial over 3‐ and 6‐month follow‐up periods.

## 2 Materials and Methods

### 2.1 Trial Design

**FIGURE 1:**
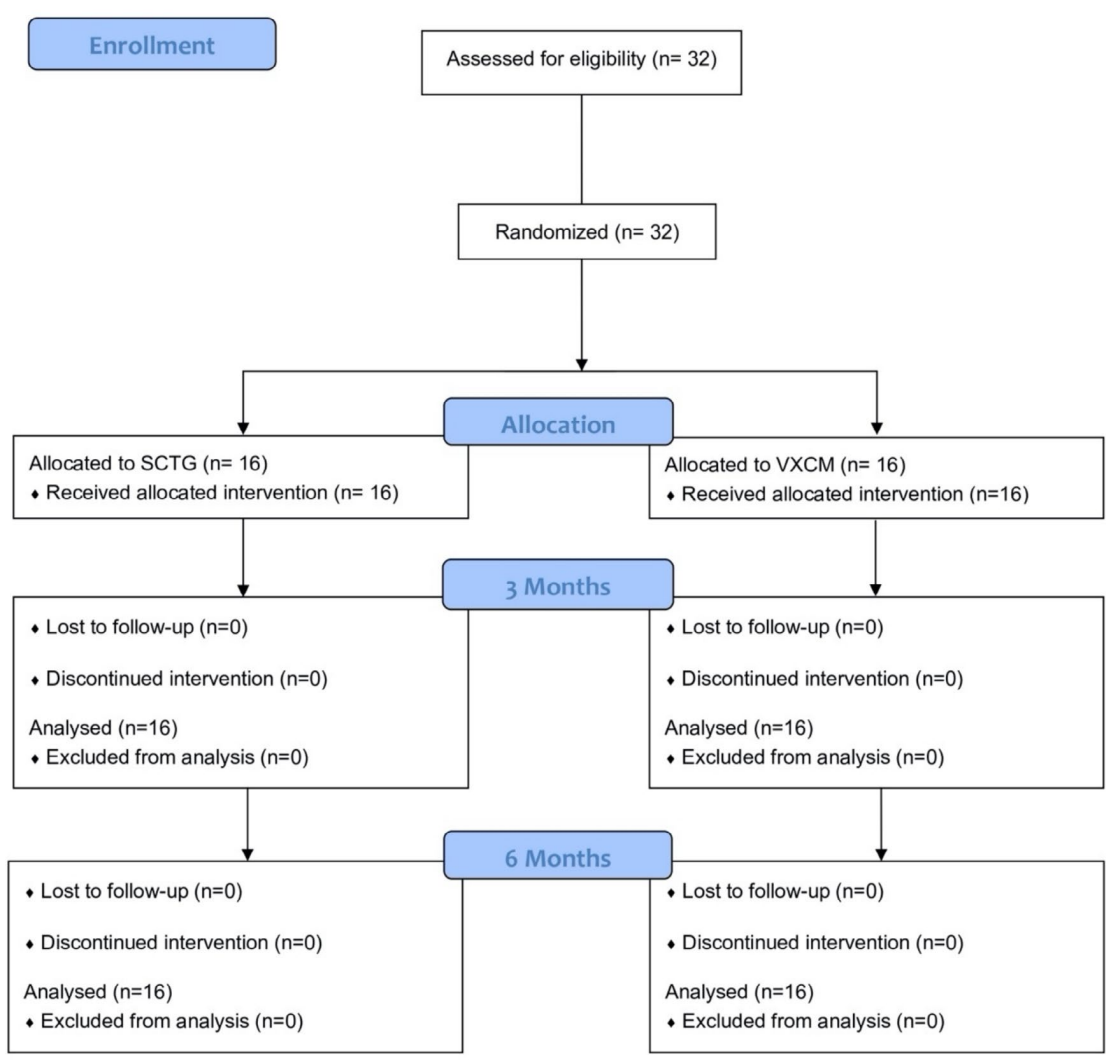
CONSORT diagram (SCTG, subepithelial connective tissue graft; VXCM, volume‐stable xenogenic collagen matrix).

This study was designed as a parallel‐arm, examiner masked, randomized controlled clinical trial, investigating the soft tissue thickness gain using VXCM and SCTG in partially edentulous patients. The study was performed in accordance with the guidelines presented in the Helsinki Declaration of Ethical Principles for Medical Research [38]. The manuscript was organized following the Consolidated Standards of Reporting Trials guidelines (CONSORT) (Figure 1). The study was authorized by the Ethical Committee (Sechenov University, Russia, Moscow), with the number 01–21/22.01.2021 and was also registered in ClinicalTrial.gov with the identifier NCT05870774. This clinical trial was not registered prior to participant recruitment and randomization. Each patient signed a written voluntary informed consent to participate in the study.

The two treatment modalities (augmentation at the implant site with SCTG and VXCX) were randomly assigned to the patients in a ratio of 1:1. The investigation aimed to detect a difference in the gain of soft tissue thickness of at least 0.3 mm between the two groups (standard deviation 0.3), retrieved from a paper published by Cairo et al. [30].

### 2.2 Treatment Modalities

- Autogenous SCTG (1 group)
- Xenogeneic VXCM (2 group)

A block randomization list was prepared using computer tables before the study started. SPSS v23 software was used for randomization. Prior to surgery, the patient and operator were blinded to the selections. Then, a pre‐prepared envelope was opened and randomized to indicate whether the patient belonged to one group or the other. Allocation concealment was performed using an envelope. A statistical analysis and a 3D analysis were conducted by blinded investigators (M.B. and A.G. respectively).

Thirty‐two patients with a single tooth gap in the posterior region of the lower jaw, combined with mucosal thickness deficiency in the area of the missing tooth, were enrolled in the study on the basis of the Department of Oral Surgery (Sechenov University, Russia, Moscow). The study recruitment began on 28.10.2021, and the last patient was treated on 25.12.2022.

### 2.3 Inclusion Criteria

- Age ≥ 18 years
- Single tooth gap (the 1st or 2nd molar on the lower jaw);
- Necessity of soft tissue augmentation at the buccal aspect;
- No previous soft tissue augmentation procedure at the operation site;
- Full‐mouth plaque score (FMPS) and full‐mouth bleeding score (FMBS) ≤ 15%.

### 2.4 Exclusion Criteria

- Heavy smokers (more than 10 cigarettes a day);
- Presence of periodontal disease;
- Necessity of bone augmentation procedures;
- Any systemic diseases that contraindicate implant placement, for example, thyroid dysfunction, autoimmune disease;
- History of malignancy, radiotherapy, or chemotherapy for malignancy over the past 5 years;
- Pregnancy and breastfeeding;
- Mental disorders;
- Allergy to collagen products.

### 2.5 Clinical Interventions

All surgeries and clinical data collection were performed by the same experienced surgeon (I.A) who has 15 years of experience after graduating from his clinical residency in oral surgery and medicine. Prior to surgery, patients received all information about possible treatment options and signed a voluntary informed consent for surgery. On the day of surgery, patients received a prophylactic dose of penicillin antibiotic (2 g amoxiclav, LEK, d.d., Slovenia) 1 h before the surgery. All patients were rinsed with 0.2% chlorhexidine solution (Corsodyl, GlaxoSmithKline) for 60 s. Local anesthesia was performed using Ubistesin Forte (articaine hydrochloride 40 mg and epinephrine hydrochloride 0.006 mg, 3 M ESPE, St. Paul, MN, USA).

The operation was performed as follows: in the area of the missing tooth, a linear incision was made along the most coronal part of the alveolar ridge, and in the area of the neighboring teeth, sulcular incisions were made. An Astra Tech dental implant (Dentsply Implants Manufacturing GmbH, Germany) was placed after raising a full‐thickness flap according to the standard surgical protocol, and healing abutments of standard size (diameter 4.5 mm, height 4 mm) were placed.

**FIGURE 2:**
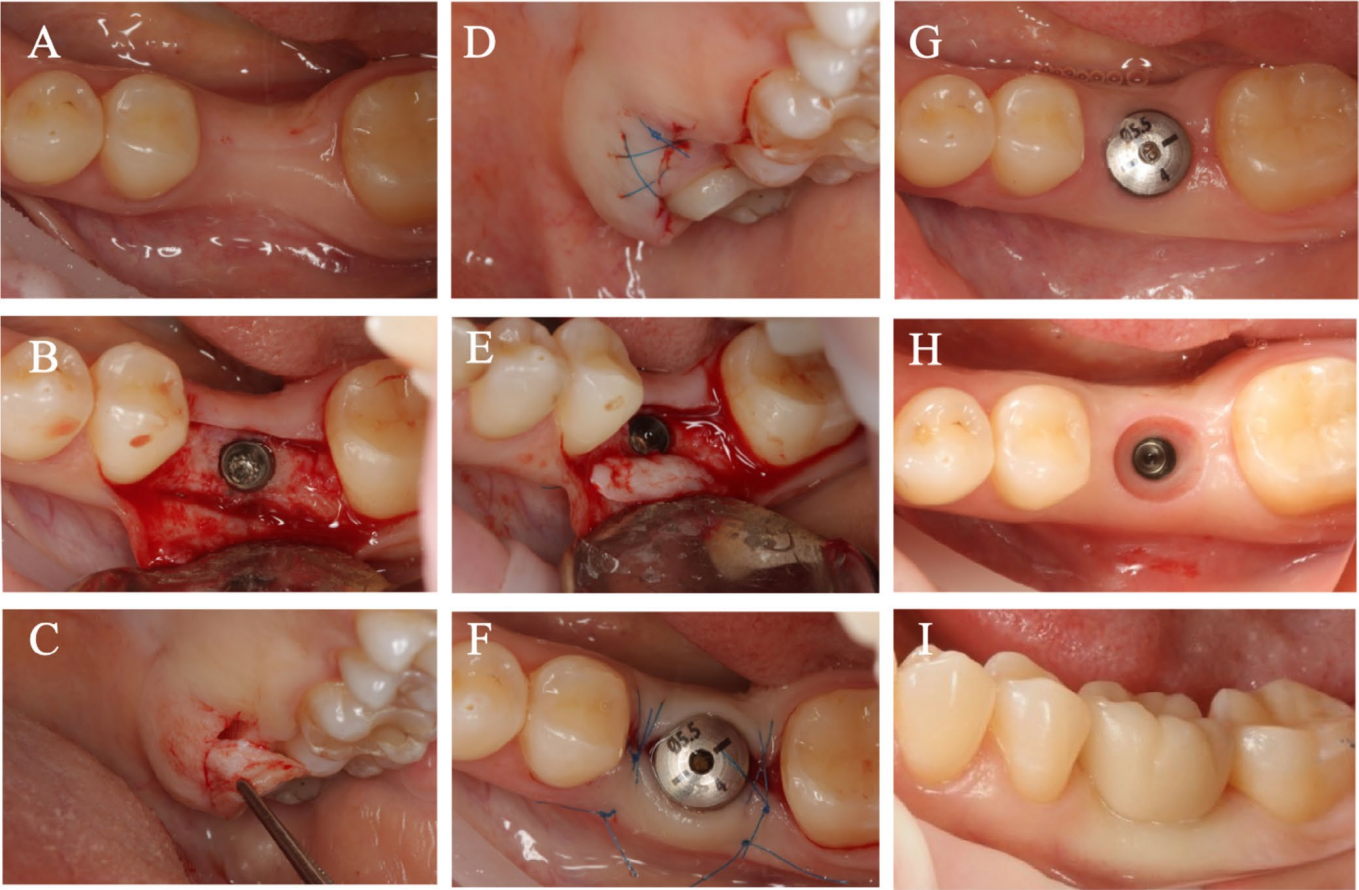
Surgical intervention in a patient allocated to the SCTG group. (A) Baseline screening. (B) Full‐thickness flap elevation and implant placement. (C) SCTG harvesting at the tuber maxilla. (D) Donor area suturing. (E) SCTG positioned under the buccal flap. (F) Wound closure and suturing around the healing abutment. (G) Follow‐up examination at 3 months. (H) Soft tissue conditions around the implant. (I) Final restoration at 6 months.

In the SCTG group, the autogenous connective tissue graft was taken from the maxillary tuberosity region using two parallel incisions. After the graft was harvested, the epithelial tissue, fat, and glandular inclusions were removed from its surface, and it was also shaped into a recipient bed. After harvesting the graft, approximating sutures were placed in the donor area. Then the graft was fixed to the buccal flap using a mattress (horizontal) suture to the buccal mucoperiosteal flap. Finally, single interrupted sutures (Prolene 6–0; Ethicon W8005, Johnson & Johnson) closed the wound around the healing abutments without any tension (Figure 2).

**FIGURE 3:**
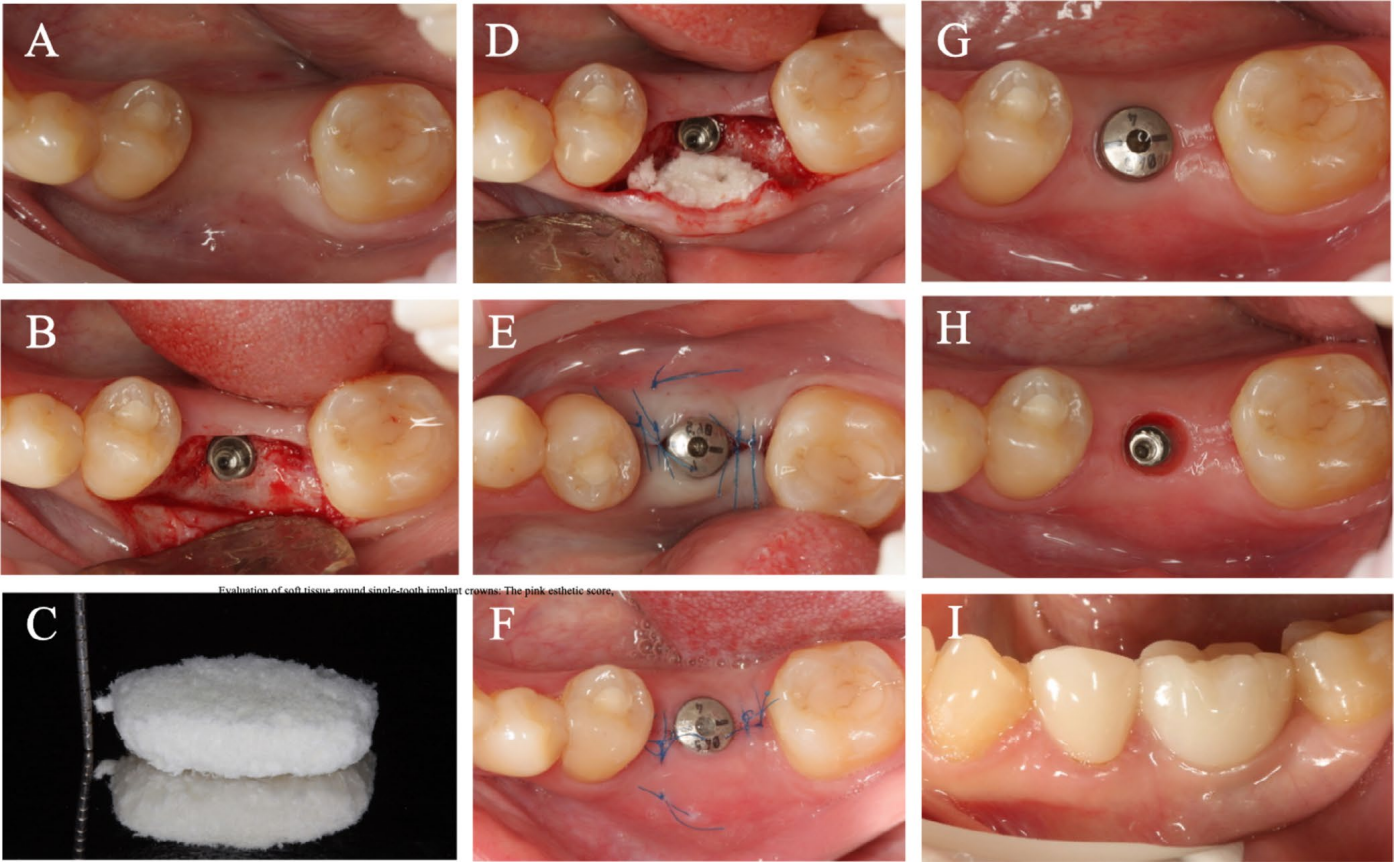
Surgical intervention in a patient allocated to the VXCM group. (A) Baseline screening. (B) Full‐thickness flap elevation and implant placement. (C) VXCM preparing. (D) VXCM positioned under the buccal flap. (E) Wound closure and suturing around the healing abutment. (F) 14 days post‐op. (G) Follow‐up examination at 3 months. (H) Soft tissue conditions around the implant. (I) Final restoration at 6 months.

In the VXCM group, the mucoperiosteal flap was mobilized by periosteum dissection. The VXCM (Fibro‐Gide, Geistlich, Switzerland) was modeled using sterile scissors according to the shape of the recipient bed and was fixed to the buccal flap in the same way (Figure 3). After that, the wound was sutured with interrupted sutures around the healing abutments. Operation time (from the first incision to the last suture) was recorded in minutes.

All patients were instructed on postoperative oral care (rinsing with 0.2% chlorhexidine solution (Corsodyl, GlaxoSmithKline) twice a day for a week and performing a daily hygiene with an extra fine toothbrush). For pain control, the patient was prescribed Nimesulide (100 mg) (Berlin Chemie Menarini, Italy). The patients were also advised to minimize trauma at the surgery site; no special diet was recommended. Two weeks after surgery, sutures were removed and patients were instructed to resume mechanical tooth cleaning. Professional oral hygiene procedures were performed at the 3‐month follow‐up.

After 3 months of healing, the restorative procedures were performed: impressions were taken using polyvinyl siloxane impression material. Final prosthetic restorations were screw‐retained. Also, periapical x‐rays were taken.

### 2.6 Evaluation of the Soft Tissue Gain

**FIGURE 4:**
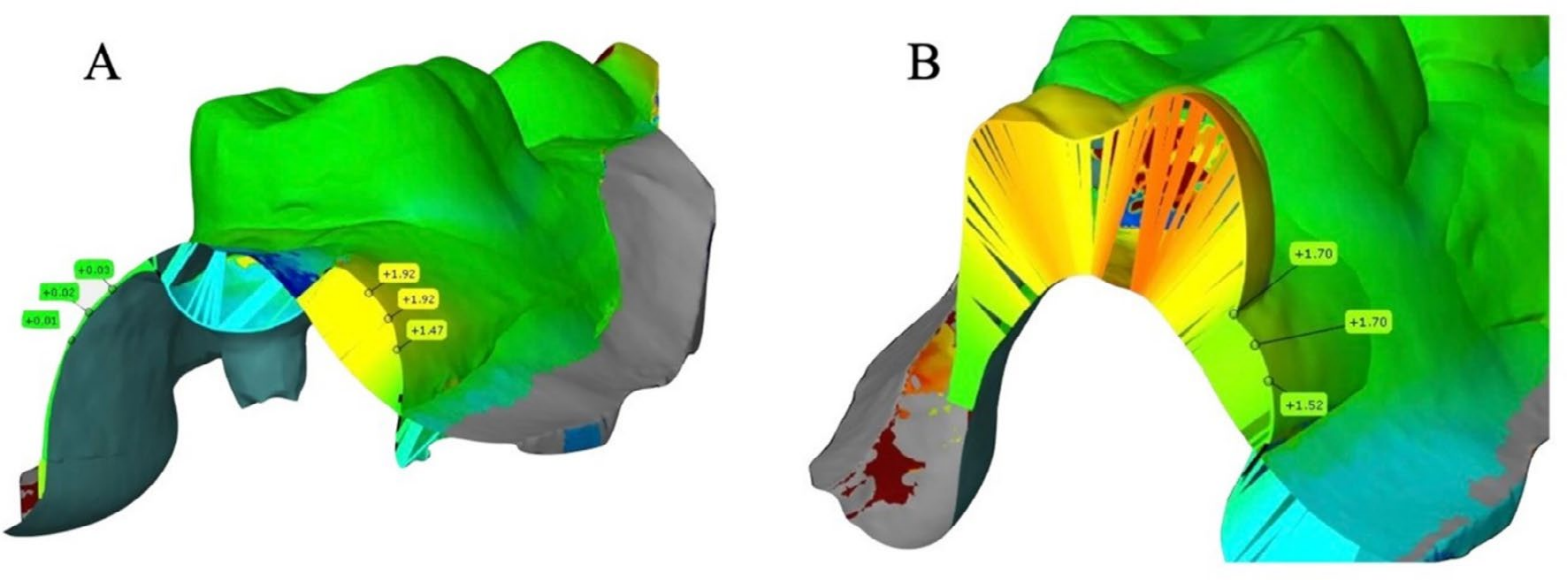
Soft tissue thickness gain: 3 (A) and 6 (B) months after the surgery.

For measuring the soft tissue gain, an optical impression was taken using an intraoral scanner (Primescan, Dentsply Sirona, Benshein, Germany), before the surgical intervention and on the 90th and 180th days after the operation. The gathered scans were exported in standard tessellation language (STL) format into metrology software (GOM Inspect, Carl Zeiss GOM Metrology GmbH, Germany). The pre‐operative scan was used as a reference. The 3‐ and 6‐month scans were matched to the reference scans using common tooth geometry. The pre‐matching was performed using a three‐point protocol, and the final matching was performed using a best‐fit protocol (assignment via individual structures within the scan). To account for soft tissue changes, the standardized region of interest (ROI) was defined. From the cross‐sectional views between the adjacent teeth (or 5 mm distally to the end of the crown if there was no distal tooth) and 1 mm apically to the buccal gingival margin. The buccal contour changes in millimeters were evaluated at 3 equidistant points at intervals of 1 mm in the coronary‐apical direction, representing the soft tissue gain (Figure 4). This 3D analysis was performed by the blinded investigator (A.G.). Before the start of the analysis, an investigator calibration was performed to determine the reproducibility of the measurements. The calibration was accepted when repeated measurements presented an intraclass correlation coefficient (ICC) < 0.85. The 3‐month soft tissue thickness gain was defined as a primary outcome. The 6‐month soft tissue thickness gain and the following clinical parameters were defined as secondary outcomes.

The scanner accuracy was calibrated before each scan using a special calibrator nozzle. The base of the nozzle was to be turned counterclockwise until it clicked 18 times, according to the instructions on the scanner screen. After 18 steps, the calibrator attachment was put on the opposite side to perform the calibration calculation.

### 2.7 Width of Keratinized Mucosa

The width of the keratinized mucosa was measured using the UNC‐15 periodontal probe. This was done by determining the mucosa‐gingival junction line on the buccal side. The distance from this line to the apex of the alveolar ridge corresponded to the width of the keratinized mucosa.

### 2.8 Severity of Pain

The evaluation of the severity of pain was carried out after surgery on 1, 3, 5, 7, 30, 90, 180 days using a standard 10‐point verbal descriptive scale of pain (Verbal Descriptor Scale) [39]; patients described their pain using a scale from 0 to 10, where 0 is “no pain” and 10 is “pain that makes you pass out.”

### 2.9 Consumption of Analgesics

The patients' analgesic consumption was assessed by estimating the number of packs of Nimesulide (100 mg) taken on 1, 3, 5, and 7 days after surgery.

### 2.10 Post‐Operative Edema/Swelling

The post‐operative edema/swelling was evaluated visually on 1, 3, 5, and 7 days after the surgery using the following scoring system: 0 = no visible edema; 1 = slight edema (intra‐oral swelling in the surgical zone); 2 = moderate edema (extra‐oral swelling in the surgical area); 3 = severe edema (extra‐oral swelling extending the surgical site) and/or visible haematoma and ecchymosis [40].

### 2.11 Quality of Life

An oral health impact profile questionnaire (OHIP‐G14) was handed out to patients and filled out at the baseline and follow‐up stages on 7, 90, and 180 days after the operation. The questionnaire consisted of 14 questions that provided a patient's self‐reported measure of dysfunction, discomfort, and disability arising from oral conditions. Each question was scored on a 5‐point scale: 0 = never; 1 = hardly ever; 2 = occasionally; 3 = fairly often; 4 = very often/every day. The OHIP‐14 total score could range from 0 to 56. Higher OHIP‐14 scores indicate a worse quality of life, and lower scores indicate a better quality of life.

### 2.12 Soft Tissue Aesthetics

The evaluation of soft tissue aesthetics was carried out by visual inspection taking into account the PES (pink aesthetic score) [41]. To assess the condition of the soft tissues surrounding the implant, a reference tooth (adjacent or on the contralateral side) was selected. Subsequently, each implant and the reference tooth were digitally photographed using a Canon EOS450D camera (Canon Inc., Japan) equipped with a Canon Macro Ring Lite MR‐14EX (Canon Inc., Japan) circular flash. The images were magnified twofold from their original size, and a comparative evaluation was conducted based on seven parameters: mesial papilla, distal papilla, soft‐tissue level, soft‐tissue contour, alveolar process deficiency, soft‐tissue color, and texture. The evaluation was carried out according to a score of 0–1‐2, where 0 was the lowest value and 2 was the highest; the maximum achievable PES value was 14. All measurements were performed by a blinded and calibrated clinician who was not involved in the surgical or prosthetic treatment. The examiner evaluated each photograph twice on different days (1 month apart).

### 2.13 Statistical Analyses

The sample size of at least 32 participants was calculated with “OpenEpi” open‐source statistical software to detect differences in the gain of soft tissue thickness of at least 0.3 mm between the two groups (standard deviation 0.3, retrieved from a paper published by Cairo) [30], using an alpha of 5% and 80% power. The sample size calculation indicated 14 patients to be included per group. To compensate for dropouts, this number was increased to 16 patients per group.

The statistical analysis was conducted by the other blinded investigator (M.B.). The GraphPad Prism v9.5.0 (GraphPad Software, USA) and R programming environment v4.1.2 (Posit PBC, USA) were used in conjunction with the *lme4* v1.1–29, *glmmTMB* v1.1.3. *emmeans* v1.7.3, *MASS* v7.3–57 libraries. The *performance* v0.9.0 library was used to check the model assumptions. For the analysis of categorical variables, frequencies were calculated, and ordered logistic regression (OLR) models were used to compare groups. For quantitative variables, descriptive statistics including mean, standard deviation (SD), median, and quartiles (Q1, Q3) were computed. The Mann–Whitney test for descriptive data and the Fisher's exact test for frequencies distribution were used to compare the volume of graft and duration of surgical intervention. Group comparisons were performed using generalized linear mixed‐effects models (GLMM), incorporating an interaction between the factors ‘group’ and ‘time’ as fixed effects, with a random intercept (ID_patient_). For soft tissue gain a random intercept was included for the observation of the factor, nested within each patient's observations. Statistical significance was set at a *p*‐value < 0.05.

## 3 Results

The patient recruitment phase started in October 2021 and ended in December 2022. In the study, 32 patients were enrolled and subjected to intervention. Sixteen patients (mean 37.19 ± 7.13 years) were allocated to group SCTG and 16 patients (mean 41.94 ± 9.62 years) to group VXCM. The groups were comparable in age and gender (*p* = 0.1989; *p* = 0.4331 respectively) (Table 1). All 32 patients included were treated, and the follow‐up was completed. The duration of surgical intervention in the SCTG and VXCM groups was 30.31 ± 6.98 (SD) and 26.31 ± 6.81 (SD) minutes, respectively (*p* = 0.1857). Regarding the volume of autograft and collagen matrix used, it was 91.3 4 ± 65.77 (SD) mm^3^ and 346.5 ± 117.99 (SD) mm^3^ in the SCTG and VXCM groups, respectively (*p* < 0.0001) (Table 1).

**TABLE 1:** Baseline characteristics.

Baseline characteristics	SCTG (*N* = 16)	VXCM (*N* = 16)
Age		
Mean (SD)	37.19 (7.13)	41.94 (9.62)
Median (Q1; Q3)	36 (32.5–40.75)	40 (33.25–51.5)
Gender		
Female	10 (62.5%)	13 (81.2%)
Male	6 (37.5%)	3 (18.8%)
Bone height, mm		
Mean (SD)	11.98 (2.05)	13.56 (1.76)
Median (Q1; Q3)	11.35 (10.18–14.58)	13.35 (12.15–15.25)
Bone width, mm		
Mean (SD)	7.17 (1.36)	8.55 (1.98)
Median (Q1; Q3)	7.25 (5.973–8.325)	8.15 (7.25–9.558)
Implant diameter		
3.5 mm	6 (37.5%)	1 (6.25%)
4 mm	7 (43.75%)	12 (75.0%)
4.2 mm	0 (0%)	1 (6.25%)
4.5 mm	3 (18.75%)	2 (12.5%)
Implant length		
9 mm	16 (100%)	13 (81.25%)
10 mm	0 (0%)	1 (6.25%)
11 mm	0 (0%)	2 (12.5%)
Torque		
Mean (SD)	40.94 (7.12)	42.19 (7.52)
Median (Q1; Q3)	45 (35–45)	40 (40–45)
Time surgery, min		
Mean (SD)	30.31 (6.98)	26.31 (6.81)
Median (Q1; Q3)	29 (23.25–37.75)	26.5 (20.25–30)
Graft volume, mm^3^		
Mean (SD)	91.34 (65.77)	346.50 (117.99)
Median (Q1; Q3)	69.75 (60–100)	318 (274.5–421.5)

### 3.1 Primary Outcomes

#### 3.1.1 Soft Tissue Thickness Gain 3 Months After Surgery

**FIGURE 5:**
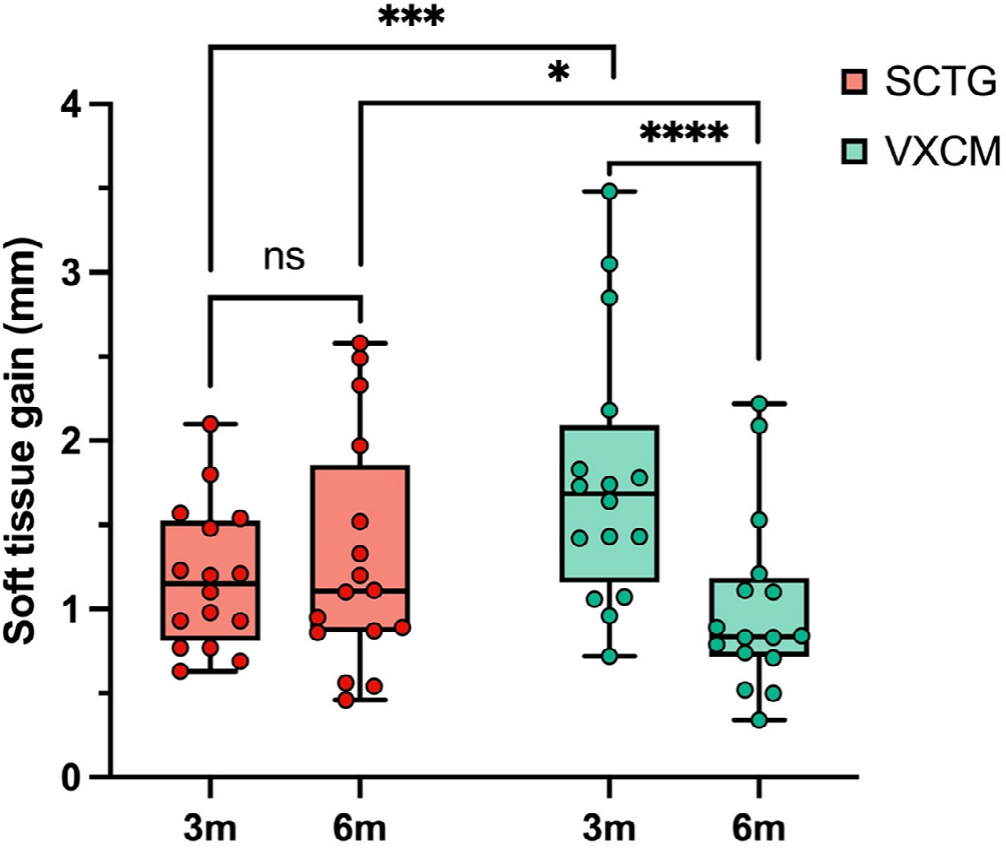
The soft tissue gain (mm) at 3 and 6 months after surgery was compared between the SCTG and VXCM groups; GLMM post hoc Sidak test was used; statistical significance *p* < 0.05 was denoted by anasterisk.

A statistically higher mean soft tissue thickness gain was observed in the VXCM group (1.77 ± 0.61 mm) after 3 months compared to the SCTG group (1.26 ± 0.41 mm) (*p* = 0.0003) (Figure 5; Table 2).

**TABLE 2:** Soft tissue thickness gain 3 and 6 months post‐operative (mm).

	SCTG (*n* = 16)	VXCM (*n* = 16)	*p*‐value (GLMM post hoc Sidak test)
Soft tissue thickness gain (mm) 3 months later			0.0003^+++^
Mean (SD)	1.26 (0.41)	1.77 (0.61)	
Median (Q1; Q3)	1.13 (0.97; 1.59)	1.66 (1.46; 2.17)	
Soft tissue thickness gain (mm) 6 months later			0.0459^+++^
Mean (SD)	1.43 (0.81)	1.11 (0.44)	
Median (Q1; Q3)	1.24 (0.73; 1.95)	1.078 (0.78; 1.20)	
Soft tissue gain by points, mm 3 months later			
Point 1, Mean (SD); Median (Q1; Q3)	1.3 (0.5); 1.33 (1.0; 1.50)	1.7 (0.6); 1.63 (1.31; 2.24)	0.074
Point 2, Mean (SD); Median (Q1; Q3)	1.29 (0.44); 1.25 (0.98; 1.67)	1.85 (0.57); 1.8 (1.55; 2.23)	**0.014**
Point 3, Mean (SD); Median (Q1; Q3)	1.18 (0.42); 1.15 (0.89; 1.50)	1.77 (0.78); 1.69 (1.33; 1.92)	**0.009**
Soft tissue gain by points, mm 6 months later			
Point 1, Mean (SD); Median (Q1; Q3)	1.57 (0.97); 1.43 (0.9; 2.04)	1.16 (0.54); 1.03 (0.74; 1.63)	0.068
Point 2, Mean (SD); Median (Q1; Q3)	1.44 (0.83); 1.31 (0.73; 1.79)	1.15 (0.45); 0.98 (0.88; 1.26)	0.208
Point 3, Mean (SD); Median (Q1; Q3)	1.3 (0.69); 1.11 (0.87; 1.63)	1.02 (0.53); 0.84 (0.73; 1.14)	0.209

*Note:* *p* value^+^ – Mann–Whitney test, *p* value^++^ − Fisher's exact test, *p* value^+++^ − GLMM post hoc Sidak test. Values that were statistically significant are highlighted in bold.

### 3.2 Secondary Outcomes

#### 3.2.1 Soft Tissue Thickness Gain 6 Months After Surgery

A statistically higher mean soft tissue thickness gain was observed in the SCTG group (1.43 ± 0.81) after 6 months compared to VXCM (1.11 ± 0.44) (*p* = 0.0459) (Figure 5, Table 2).

#### 3.2.2 Width of Keratinized Mucosa

**FIGURE 6:**
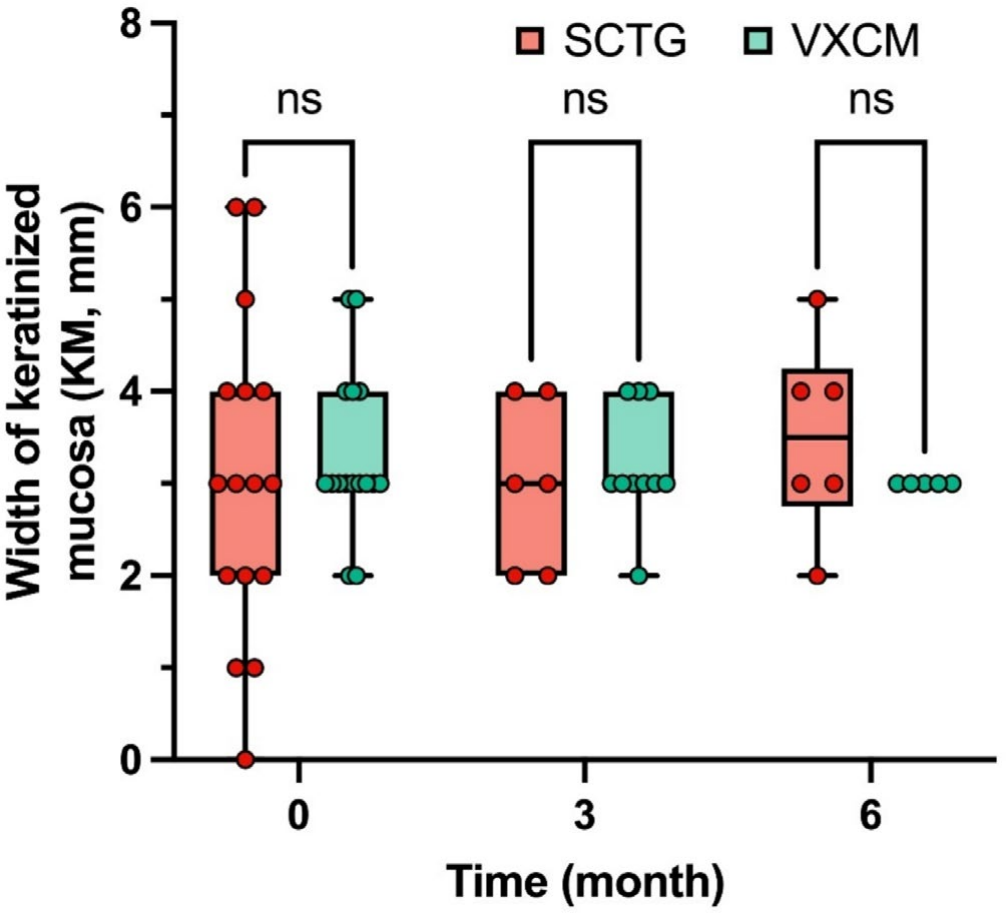
The width of keratinized mucosa on the buccal side before surgery, 3 and 6 months after surgery, between groups SCTG and VXCM; GLMM post hoc Sidak test was used; no statistical difference was observed.

No statistically significant difference was observed between the SCTG and VXCM groups in the width of the keratinized mucosa on the buccal side preoperatively (3.06 ± 1.73 and 3.31 ± 0.87 mm) and postoperatively (3.20 ± 0.63 mm and 3.00 ± 0.89 mm) after 3 months, and (3.50 ± 1.05 and 3.00 ± 0.00 mm) after 6 months, respectively (*p* = 0.082) (Figure 6, Table 3).

**TABLE 3:** Amount of width of keratinized mucosa (KM) (mm).

Time	SCTG (*n* = 16) Mean (SD); Median (Q1; Q3)	VXCM (*n* = 16) Mean (SD); Median (Q1; Q3)	*p*‐value (GLMM post hoc Sidak test)
Before surgery	3.06 (1.73); 3.0 (2.0; 4.0)	3.31 (0.87); 3.0 (3.0; 4.0)	0.604
3 months	3.20 (0.63); 3.0 (2.0; 4.0)	3.0 (0.89); 3.0 (3.0; 4.0)	0.657
6 months	3.50 (1.05); 3.50 (2.75; 4.25)	3.0 (0.0); 3.0 (3.0; 3.0)	0.082

#### 3.2.3 Severity of Pain

**FIGURE 7:**
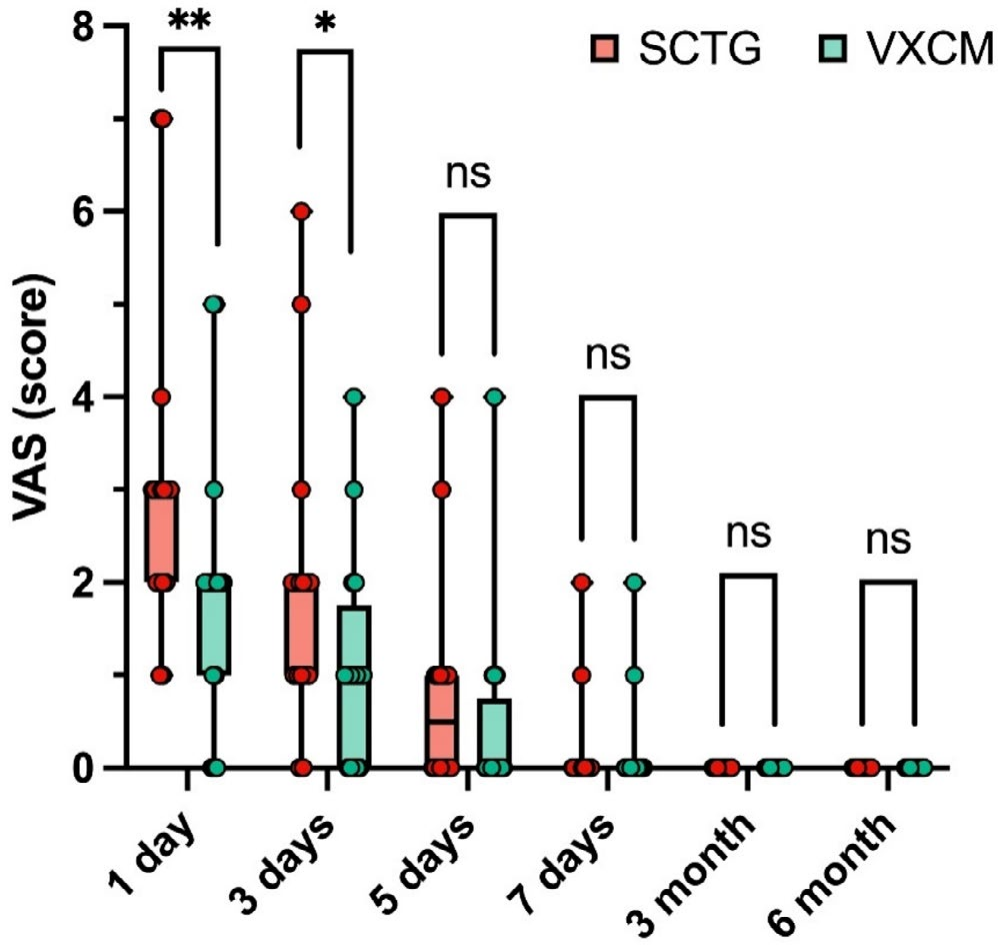
Pain score (VAS) between SCTG and VXCM after 1, 3, 5, 7 days, 3 and 6 months; GLMM post hoc Sidak test was used; statistical significance *p* < 0.05 was denoted by anasterisk.

Statistically higher pain severity was observed using the VAS scale in the SCTG group (3.06 ± 1.73) compared to the VXCM group (1.94 ± 1.48) on the 1st day after surgery (*p* = 0.002). Statistically higher pain severity was observed using the VAS scale in the SCTG group (1.94 ± 1.61) compared to the VXCM group (1.13 ± 1.15) on the 3rd day after surgery (*p* = 0.024). Starting from the 7th day, no significant differences were observed between both groups. After 3 and 6 months, patients did not experience discomfort in the surgical area (Figure 7, Table 4).

**TABLE 4:** Pain score (VAS).

Time	SCTG (*n* = 16) Mean (SD); Median (Q1; Q3)	VXCM (*n* = 16) Mean (SD); Median (Q1; Q3)	*p*‐value (GLMM post hoc Sidak test)
1 day	3.06 (1.73); 3.0 (2.0; 3.0)	1.94 (1.48); 2.0 (1.0; 2.0)	0.002
3 days	1.94 (1.61); 2.0 (1.0; 2.0)	1.13 (1.15); 1.0 (0.0; 1.75)	0.024
5 days	0.81 (1.17); 0.5 (0.0; 1.0)	0.44 (1.03); 0.0 (0.0; 0.75)	0.294
7 days	0.19 (0.54); 0.0 (0.0; 0.0)	0.19 (0.54); 0.0 (0.0; 0.0)	1.000
3 months	0.0 (0.0); 0.0 (0.0; 0.0)	0.0 (0.0); 0.0 (0.0; 0.0)	1.000
6 months	0.0 (0.0); 0.0 (0.0; 0.0)	0.0 (0.0); 0.0 (0.0; 0.0)	1.000

#### 3.2.4 Consumption of Analgesics

**FIGURE 8:**
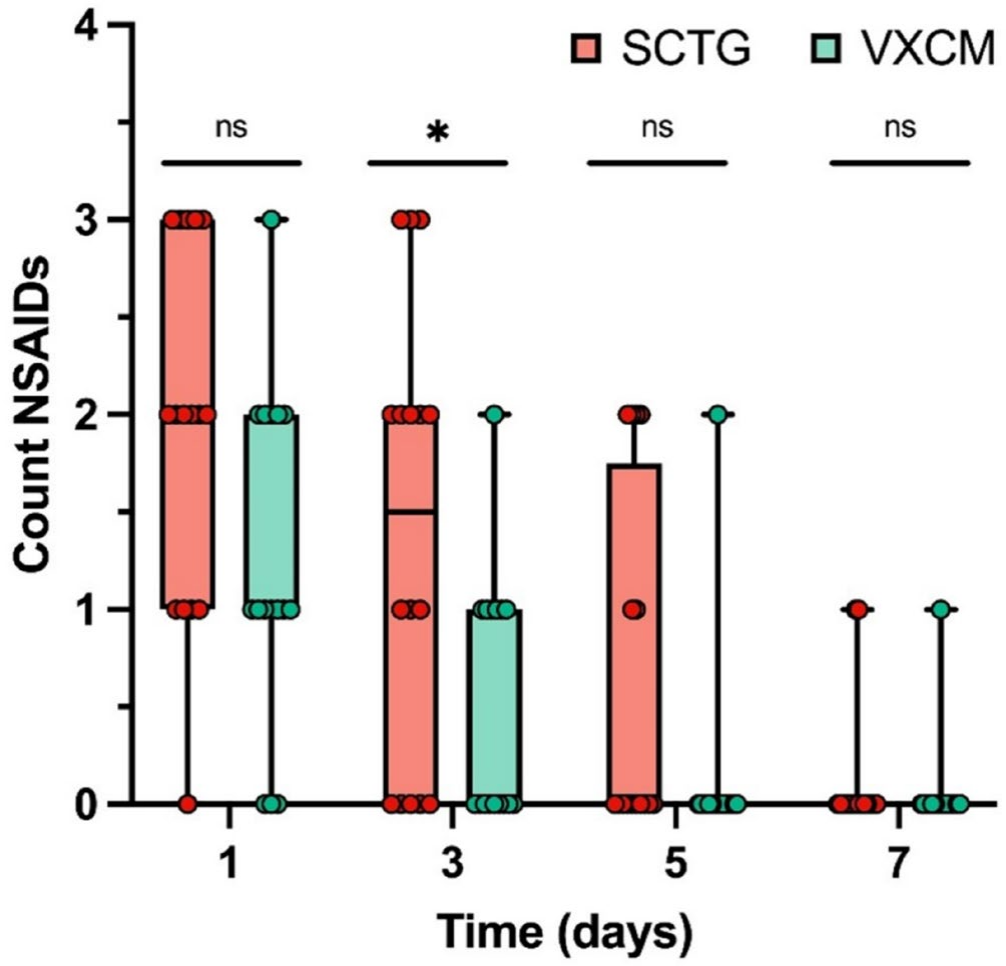
Amount of consumption of analgesics (number of packs of Nimesulide 100 mg) between SCTG and VXCM at 1, 3, 5, and 7 days after the surgery; GLMM post hoc Sidak test was used; statistical significance *p* < 0.05 was denoted by asterisk.

Statistically higher NSAID consumption was observed on the 3rd day after surgery in the SCTG group (1.50; Q1 0.00, Q3 2.00) compared with the VXCM group (0.00; Q1 0.00, Q3 1.00) (*p* = 0.027) (Figure 8, Table 5).

**TABLE 5:** The amount of NSAIDs post‐operative (packs).

Time	SCTG (*n* = 16) Mean (SD); Median (Q1; Q3)	VXCM (*n* = 16) Mean (SD); Median (Q1; Q3)	*p*‐value (GLMM post hoc Sidak test)
1 day	1.94 (0.93); 2.00 (1.0; 3.0)	1.25 (0.86); 1.0 (1.0; 2.0)	0.205
3 days	1.375 (1.15); 1.50 (0.0; 2.0)	0.5 (0.63); 0.0 (0.0; 1.0)	0.027
5 days	0.63 (0.89); 0.0 (0.0; 1.25)	0.13 (0.5); 0.0 (0.0; 0.0)	0.043
7 days	0.13 (0.34); 0.0 (0.0; 0.0)	0.06 (0.25); 0.0 (0.0; 0.0)	0.570

#### 3.2.5 Post‐Operative Edema/Swelling

**FIGURE 9:**
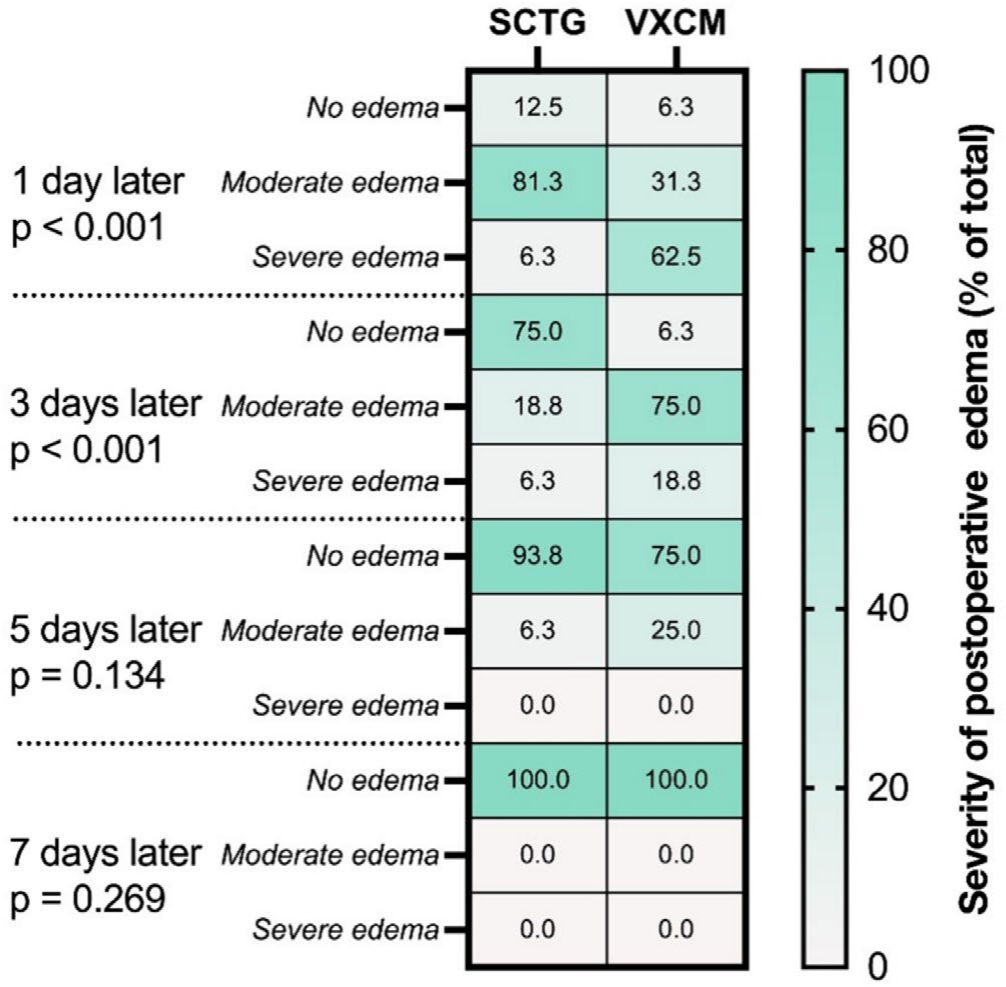
Severity of postoperative edema (frequency, % of total in group) between SCTG and VXCM at 1, 3, 5, 7 days after surgery, OLR; *p* value is significant < 0.05.

A statistically significant edema was observed on the 1st day after surgery in the SCTG group compared to the VXCM group (81.3% and 18.75% of patients respectively) (*p* < 0.001). However, on the 3rd day, a statistically significant edema was demonstrated by the VXCM group (75% in contrast to 18.8% by SCTG) (*p* < 0.001) (Figure 9, Table 6).

**TABLE 6:** Severity of postoperative edema/swelling (% of total).

Time	Range	SCTG (*n* = 16)	VXCM (*n* = 16)	*p*‐value (OLR)
1 day	No edema	2 (12.5%)	1 (6.25%)	< 0.001
	Moderate edema	13 (81.25%)	5 (31.25%)	
	Severe edema	1 (6.25%)	10 (62.5%)	
3 days	No edema	12 (75%)	1 (6.25%)	< 0.001
	Moderate edema	3 (18.75%)	12 (75%)	
	Severe edema	1 (6.25%)	3 (18.75%)	
5 days	No edema	15 (93.75%)	12 (75%)	0.134
	Moderate edema	1 (6.25%)	4 (25%)	
	Severe edema	0 (0%)	0 (0%)	
7 days	No edema	16 (100%)	16 (100%)	0.269
	Moderate edema	0 (0%)	0 (0%)	
	Severe edema	0 (0%)	0 (0%)	

#### 3.2.6 Quality of Life

**FIGURE 10:**
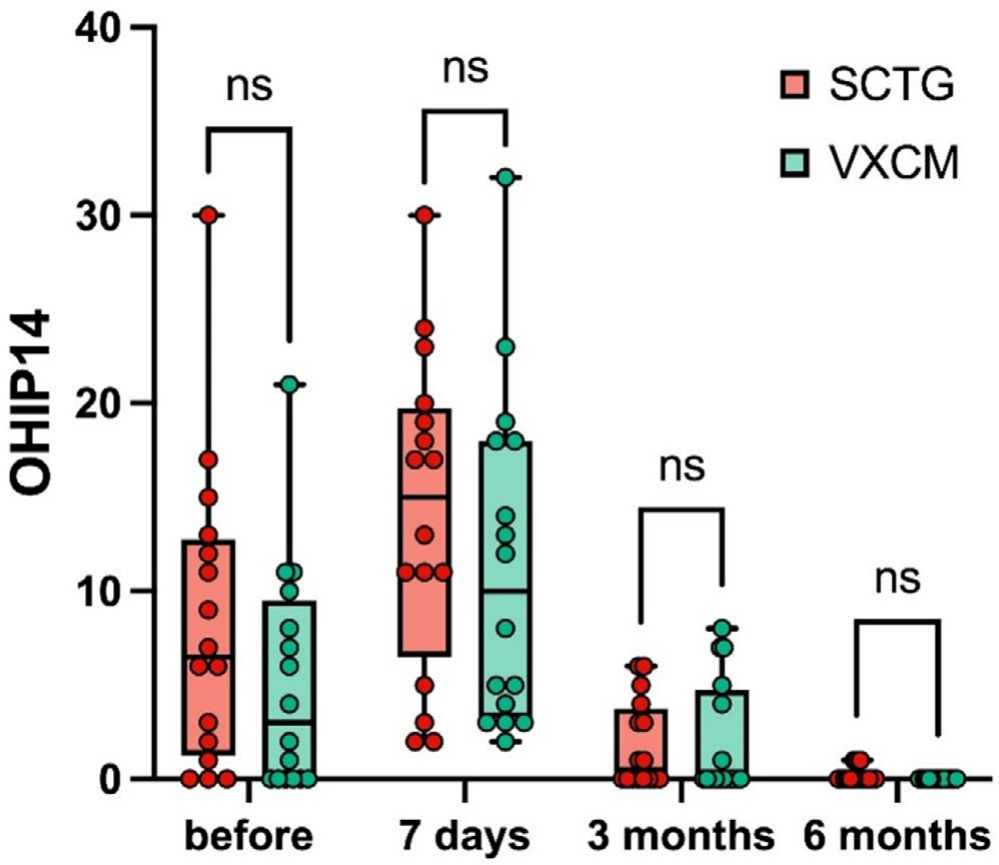
Quality of life (OHIP14 score) between SCTG and VXCM preoperatively (before), 7 days, 3, and 6 months after surgery; GLMM post hoc Sidak test was used; no statistical difference was observed.

No statistical difference between both, SCTG and VXCM groups, was observed in the quality of life (*p* = 0.282) (Figure 10, Table 7).

**TABLE 7:** Assessment of the quality of life (OHIP14 score).

Time	SCTG (*n* = 16) Mean (SD); Median (Q1; Q3)	VXCM (*n* = 16) Mean (SD); Median (Q1; Q3)	*p*‐value (GLMM post hoc Sidak test)
Before surgery	8.25 (8.06); 6.0 (1.25; 12.75)	5.06 (6.02); 3.0 (0.0; 9.5)	0.282
7 days	14.13 (8.37); 15.0 (6.5; 19.75)	11.38 (8.8); 10.0 (3.25; 18.0)	0.081
3 months	1.81 (2.32); 0.5 (0.0; 3.75)	2.0 (3.06); 0.0 (0.0; 4.75)	0.721
6 months	0.19 (0.40); 0.0 (0.0; 0.0)	0.0 (0.0); 0.0 (0.0; 0.0)	0.620

#### 3.2.7 Soft Tissue Aesthetics

**FIGURE 11:**
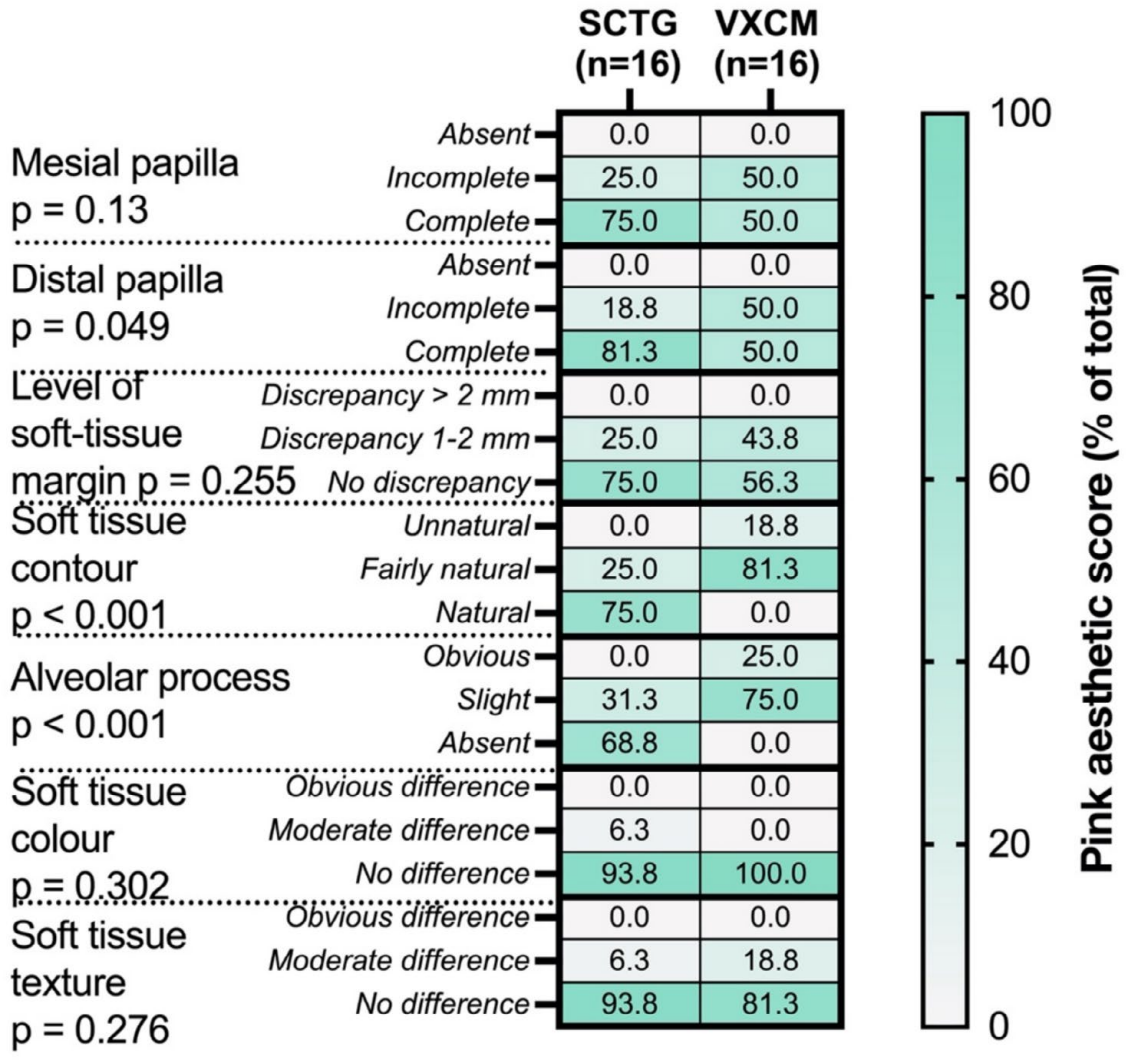
Soft tissue aesthetics score (frequency, % of total in group) between SCTG and VXCM, OLR; *p* value is significant < 0.05.

With regards to “pink aesthetics”, the SCTG group demonstrated superior results compared to the VXCM group (Total PES *p* < 0.001) (Table 8). 25% of patients in the VXCM group had obvious and 75% had slight alveolar process deficiency, whereas in the SCTG group, the majority (68.75%) of patients had no alveolar process deficiency (*p* < 0.001) (Figure 11). With regards to the soft tissue contour, the SCTG group demonstrated higher aesthetics than the VXCM group (*p* < 0.001). No statistical differences were observed with regards to mesial papilla, the level of soft‐tissue margin, soft tissue color, or soft tissue texture.

**TABLE 8:** Soft tissue aesthetics (pink aesthetic score).

Area	Group	Range			Mean score (95% CI)	*p*‐value (OLR)
*Mesial papilla*		*Absent*	*Incomplete*	*Complete*		0.131
SCTG (*n* = 16)		0 (0%)	4 (25%)	12 (75%)	2.75 (2.54–2.96)	
VXCM (*n* = 16)		0 (0%)	8 (50%)	8 (50%)	2.5 (2.25–2.74)	
*Distal papilla*		*Absent*	*Incomplete*	*Complete*		0.049
SCTG		0 (0%)	3 (18.75%)	13 (81.25%)	2.81 (2.62–3)	
VXCM		0 (0%)	8 (50%)	8 (50%)	2.5 (2.25–2.74)	
*Level of soft‐tissue margin*		*Major discrepancy* (> *2 mm*)	*Minor discrepancy* (*1–2 mm*)	*No discrepancy* (< *1 mm*)		0.255
SCTG		0 (0%)	4 (25%)	12 (75%)	2.75 (2.54–2.96)	
VXCM		0 (0%)	7 (43.75%)	9 (56.25%)	2.56 (2.32–2.81)	
*Soft tissue contour*		*Unnatural*	*Fairly natural*	*Natural*		< 0.001
SCTG		0 (0%)	4 (25%)	12 (75%)	2.75 (2.54–2.96)	
VXCM		3 (18.75%)	13 (81.25%)	0 (0%)	1.81 (1.62–2)	
*Alveolar process*		*Obvious*	*Slight*	*None*		< 0.001
SCTG		0 (0%)	5 (31.25%)	11 (68.75%)	2.69 (2.46–2.91)	
VXCM		4 (25%)	12 (75%)	0 (0%)	1.75 (1.54–1.96)	
*Soft tissue color*		*Obvious difference*	*Moderate difference*	*No difference*		0.302
SCTG		0 (0%)	1 (6.25%)	15 (93.75%)	2.94 (2.82–3.06)	
VXCM		0 (0%)	0 (0%)	16 (100%)	3 (3–3)	
*Soft tissue texture*		*Obvious difference*	*Moderate difference*	*No difference*		0.276
SCTG		0 (0%)	1 (6.25%)	15 (93.75%)	2.94 (2.82–3.06)	
VXCM		0 (0%)	3 (18.75%)	13 (81.24%)	2.81 (2.62–3)	
Total PES	SCTG (*n* = 16)				11.86 (11.13–12.59)	< 0.001
	VXCM (*n* = 16)				9.17 (8.19–10.15)	

## 4 Discussion

In the presented study, both materials resulted in an increase in soft tissue thickness compared to baseline. The greatest increase was observed in the VXCM group, and there was no statistically significant difference between the groups at the 3‐month follow‐up. The findings are consistent with the study conducted by Thoma et al., in which the authors also demonstrated greater buccal soft tissue thickness gains in the VXCM group compared to the SCTG group [20]. Simultaneously, a number of studies reveal either comparable [31, 42] or lesser [32] increases in soft tissue thickness when employing VXCM as opposed to SCTG at the benchmark of 3 months after augmentation.

The discrepancy in findings at the 3‐month follow‐up of the present study compared to those of other researchers can be attributed to methodological differences. In the current study, the augmentation of soft tissues was conducted during single‐stage implantation. In contrast, similar studies have involved soft tissue augmentation at the implant placement stage with complete, tight wound closure [42]; 1.5–6 months after implant placement, but prior to its exposure [31]; or at the implant placement stage with the immediate installation of a provisional crown [32]. The varying positions of the covering flap may have influenced the outcome.

After 6 months, however, the opposite result was observed: statistically significant soft tissue increases were observed in the SCTG group compared to the VXCM group, possibly due to the ongoing shrinkage of the VXCM. Moreover, the greater increase when using SCTG after 6 months compared to the values after 3 months can be explained by the displacement of soft tissues by permanent crown pressure since the final measurement was carried out after the final implant crown placement. Thus, we cannot be sure that the changes that occurred reflect the actual condition of soft tissue thickness, and the obtained information allows us to understand only a certain tendency.

The hypothesis regarding the ongoing resorption of VXCM is confirmed in the study by Cosyn et al., wherein the authors observe continued shrinkage in both the VXCM and the CTG application site 1 year compared to the outcome at 3 months post‐operatively [33]. It is important to note that in their investigation, the authors used CTG from the hard palate, which may be associated with a significant shrinkage of the autogenous graft, as opposed to the findings in the current study, where CTG was harvested from the maxillary tuberosity region.

The advantages of grafts from the maxillary tuberosity compared to the lateral palate are not solely attributed to the increased thickness of soft tissues in this area, but also to the absence of fatty and glandular inclusions, rendering the tuberosity grafts more resistant to shrinkage [43]. However, recent publications suggest that under certain circumstances, these grafts may induce hyperplastic reactions in the augmentation site [35, 36].

The findings are also consistent with recent systematic reviews, which demonstrated the superiority of a connective tissue graft compared to the collagen matrix [44, 45, 46].

It is important to note that in our study, we conducted augmentation of soft tissues with a single‐stage installation (with a healing abutment), which implies the potential for contact between CTG or VXCM and the external environment. It is known that a collagen matrix is designed to heal in a submerged environment. In the event of exposure, the resulting increased remodeling processes can lead to the resorption of the matrix. A similar phenomenon was observed by Hämmerle et al. in cases of partial suture divergence on the occlusal surface, leading to a more pronounced difference in growth compared to SCTG [31]. It can be hypothesized that in our case, a more pronounced resorption of the collagen matrix also occurred, which affected the long‐term outcome.

It must be emphasized that measuring the increase in soft tissue thickness is associated with certain difficulties and limitations. Various methods for measuring soft tissue thickness have been described in the existing literature on this subject. For example, Thoma et al. assessed mucosal thickness using an endodontic instrument with a silicone stopper and an individualized stent with three standardized openings, and Cairo et al. conducted measuring using an injection needle [20, 30]. Also, described is a method for measuring the soft tissue thickness by puncturing the mucosa with a periodontal probe within the attached mucosa 1–2 mm apically to the bottom of the gingival sulcus [47]. These methods are invasive, are accompanied by additional pain, and cause certain difficulties when carried out at stages that do not involve surgical treatment. The use of ultrasound devices and digital profilometry are distinguished among non‐contact and less traumatic methods for assessing the soft tissue volume [18, 48]. To assess soft tissue thickness in the present study, we used a 3D analysis of STL files obtained from intraoral scanning. The benefit of using digital methods is that there is a reduction in the number of possible errors in measurements, an increase in the reliability of the method, and high reproducibility of results [6, 49].

It is known that the resorption of the buccal bone after tooth extraction leads to the invagination of the alveolar ridge contour. These deformations occur not only in the esthetically significant areas but also in the posterior regions [4, 5, 6]. There is some evidence that immediate implantation in the aesthetic area should be coupled with socket preservation and a soft tissues augmentation procedure. This approach makes it possible to compensate for the deformation of the buccal contour [50, 51]. The soft tissue augmentation using SCTG or VXCM in a healed socket allows correcting the buccal contour, highlighting the significance of the soft tissue component in achieving an aesthetic outcome [18, 19]. Although in the present study the aesthetic results were satisfactory in both groups, the results in the VXCM group were inferior in some respects to the outcomes in the SCTG group. This indirectly confirms the greater effectiveness of using SCTG when assessed 6 months after surgery. On the contrary, the study by Cosyn et al. reveals no significant differences between the groups in terms of patients' aesthetic satisfaction with the treatment performed and the PES scores 1 year post‐operatively [33]. This finding is corroborated by a recent systematic review conducted by Thoma et al. and a multicenter study led by Hämmerle et al. [31, 34]. It is worth noting, however, that the latter study pertains to short‐term (3‐month) outcomes and necessitates longer‐term observations due to the ongoing shrinkage.

There are no data regarding the optimal horizontal thickness of soft tissues for the restoration of a natural contour of the alveolar ridge, as this parameter is individual and subject to bone remodeling. There are also no data to determine how important it is to achieve good aesthetic results in the posterior region of the jaws. However, in recent years there have been increasingly more patients, especially younger ones, for whom this is really important.

At 3 and 6 months after surgery, the greatest increase in the keratinized attached mucosa width at the buccal aspect was observed in the SCTG group, but there was no statistically significant difference between the groups. The findings are consistent with the study by Hélio et al., where the authors also noted the greatest increase in the KMW at 3 months after surgery in the SCTG group, but the difference between groups was not statistically significant [52]. The 6‐month data from the present study can also be compared with the results found in Cairo et al., where the authors found a statistically significant increase in the KMW relative to the initial KMW in both groups, but no significant difference in gain was determined between the groups [30]. Similar results were also found in the study by Baldi et al., who analyzed the results after 6 months and found no statistical difference between VXCM and SCTG groups, while both treatment modalities showed a significant increase in the KMW compared to the baseline [37].

In the present study, the surgical time required for surgery using VXCM was less than that required for surgery with SCTG, due to the lack of a second surgical site. Despite this, the difference between the groups was not statistically significant, which may be due to the fact that additional time was spent preparing the VXCM (forming the required size). This is also supported by a systematic review conducted by Valles et al. [53]. On the other hand, Thoma et al. showed a reduction in operation time between the two treatment modalities [34]. There is an opinion that the use of VXCM can reduce operative time compared to the use of SCTG, only after receiving specialized training in the preparation and handling of soft tissue substitutes [46].

In the present study, patients also reported more severe postoperative pain in the early postoperative period after using SCTG, which was also confirmed by a higher consumption of NSAIDs. However, at the follow‐up stage, there was no difference between the groups. There are varying findings in the literature regarding the difference in postoperative pain between SCTG and VXCM [34]. Thus, a number of studies, including systematic reviews, report more pronounced postoperative discomfort in patients who underwent SCTG harvesting [30, 31, 34, 42]. On the other hand, other studies demonstrate that pain perception does not differ significantly between SCTG and soft tissue substitutes [32, 53]. It should be noted that the severity of postoperative discomfort and pain depends on the location of the donor area and the technique of SCTG harvesting [54]. The lack of difference in postoperative morbidity in the present study is explained by more comfortable healing after SCTG harvesting from the maxillary tuberosity area compared to the hard palate. For example, in the study by De Angelis et al. [42] SCTG was harvested using a method that involves an open wound in the hard palate that heals by secondary intention [42]. It is clear that in this case, postoperative pain in the VXCM group was lower than in the SCTG group.

Postoperative swelling in our study was more severe in patients treated with VXCM. In our opinion, this could be a consequence of the necessity to mobilize the mucoperiosteal flap for suturing without tension, which is due to the large volume of VXCM used compared to SCTG. The formation of a more pronounced edema in patients after VXCM transplantation could also be the reason for the decreased quality of life in patients in this group, although the difference was not statistically significant.

Despite the fact that, according to a number of authors of clinical studies and systematic reviews, the use of VXCM leads to a decrease in the consumption of painkillers, a reduction in the time of surgical intervention, and is characterized by a high degree of patient satisfaction with the result of treatment, this study only partially confirms these statements [30, 31, 34, 42]. Thus, according to the data of this study, the difference in the duration of the procedure between the groups was not significant. Post‐operative pain when using SCTG was more pronounced only on the first day after surgery, while in the VXCM group patients noted a more pronounced edema and impaired quality of life. However, all treated patients were satisfied with the final aesthetic results, with minor differences in favor of SCTG.

A correct interpretation of the present study necessitates consideration of the following limitations. Firstly, in order to determine the clinical efficacy of both augmentation procedures, patients need to be followed for a longer period. Secondly, the study is characterized by a small sample size and a lack of statistical power, which could have also contributed to misleading information. Thirdly, the presence of a restoration on the implant renders the results of a comparison of 3D objects at 6 months post‐surgery not entirely reliable, as the restoration may cause soft tissue displacement.

This RCT provides data regarding the use of VXCM in single‐stage implantation. Despite the limitations mentioned above, the use of VXCM demonstrates efficacy and safety. On the assumption that patients prefer a less painful and more rapid procedure, and clinicians want to simplify the surgery, the use of collagen matrices seems to be an acceptable alternative to SCTG for increasing soft tissue thickness in the area of dental implants. However, it is important to discuss with the patient the possible scope of rehabilitation in terms of aesthetics. The present results may help clinicians in the decision‐making process in their clinical practice.

## 5 Conclusion

The present randomized controlled study demonstrates that the use of VXCM may provide comparable clinical outcomes to the use of SCTG, as the soft tissue thickness gain on the 3rd month was significantly higher in the VXCM group. However, the final assessment of the treatment result should be carried out starting from the 6th month after surgery due to the ongoing shrinkage of the material, and it was in the 6th month that SCTG demonstrated a significantly higher soft tissue thickness gain. On the other side, the use of VXCM reduces the severity of postoperative pain and analgesic consumption while at the same time providing acceptable aesthetic results.

## Author Contributions

Conceptualization: I.A., S.T. and A.U.; formal analysis: M.B.; investigation: I.A., M.M., A.G.; resources: S.T.; data curation: N.S., S.K.; writing – original draft preparation: I.A., A.G., S.T. and A.U.; writing – review and editing: A.U., I.A.; visualization: A.U. and A.G.; supervision: S.T.; and project administration: I.A.

## Conflicts of Interest

The authors declare no conflicts of interest.

## Data Availability

The data that support the findings of this study are available from the corresponding author upon reasonable request.
